# Immunohistochemical expression of interleukin 8 in skin biopsies from patients with inflammatory acne vulgaris

**DOI:** 10.1186/1746-1596-2-4

**Published:** 2007-01-30

**Authors:** Howayda S Abd El All, Noha S Shoukry, Rabee A El Maged, Mostafa M Ayada

**Affiliations:** 1 Department of Pathology, Faculty of Medicine, Suez Canal University, Ismailia, Egypt; 2 Department of Dermatology and Andrology Faculty of Medicine, Suez Canal University, Ismailia, Egypt

## Abstract

**Background:**

This study was conducted to evaluate the immunohistochemical (IHC) expression of interleukin 8 (IL-8) in skin biopsies of inflammatory acne vulgaris (IAV) in an attempt to understand the disease pathogenesis.

**Materials and methods:**

A total of 58 biopsies, 29 from lesional IAV and 29 normal non lesional sites were immunostained for IL-8. The intensity of staining was evaluated in the epidermis and dermis and was scored as mild, moderate and severe. The expression was correlated with the clinical grade, disease course and histological changes.

**Results:**

IL-8 immunoreactivity was expressed in lesional IAV compared to non lesional skin biopsies (p < 0.001). Increased expression was significantly associated with epidermal hyperplasia and follicular hyperkeratosis (p < 0.001). In addition, the more pronounced IL-8 expression of the dermal endothelial cells and neutophilic inflammatory infiltrate correlated with dermal angiogenesis and the extent of dermal inflammatory response (p < 0.001). Moreover, increased dermal immunoreactivity paralleled progressive course (p = 0.02) but not the duration of the disease.

**Conclusion:**

We were able to demonstrate altered immunoreactivity of IL-8 in IAV compared to normal skin. Targeted therapy to block IL-8 production may hold promise in limiting the deleterious effects of IL-8-mediated inflammatory response and angiogenesis.

## Background

Acne vulgaris is a chronic inflammatory disease of the pilosebaceous units characterized by the formation of comedones, erythrematous papules and pustules, less frequently by nodules or pseudocyts [1]. It is a pleomorphic disorder with multifactorial pathogenesis [2]. Acne has a significant economic and social impact as well as a negative effect on self-image and outlook, especially during the emotionally critical period of adolescence [3]. Propionibacterium acnes (P. acnes), an anaerobic pathogen plays an important role in the pathogenesis by triggering the proinflammatory mediators [4,5] through activation of Toll-like receptors 2 (TLR2) [6-8]. Among these mediators, IL-8 originally identified as neutrophil-activating peptide-1 [9], along with P. acnes induce chemotactic factors that play a role in attracting neutrophils to the pilosebaceous unit (6, 7, 8). The production of IL-8 by P. acnes is through activation of the NF-kappa B [10]. Gene array expression profiling in acne lesions reveals marked upregulation of genes, including IL-8, involved in inflammation and matrix remodelling [11].

To our knowledge, no reports evaluated the IL-8 IHC expression in skin biopsies of inflammatory acne. Therefore, this study was conducted to assess this expression and to correlate it with disease severity and histological changes in an attempt to understand the disease pathogenesis. The elucidation of this role may highlight the potential role of IL-8 in therapeutic targets in inflammatory acne.

## Materials and methods

### Patients

This study is a case-control-study involving 58 skin specimens divided into two groups. The first group involved 29 specimens from patients suffering from IAV and the second one 29 from non lesional skin of same patient, used as a control. Acne severity was graded as mild, moderate and severe according to the American Academy of Dermatology Consensus statement on acne classification [12]. Four to five mm skin biopsies were taken from the papular lesion after obtaining patients' consent. Patients were ≥ 15 years old untreated or their treatment was stopped for at least two months before the biopsies, and without systemic or other inflammatory skin diseases. Complete medical history, family history of acne and previous treatment received were assessed.

### Biopsies and pathological examination

Biopsies were taken under local anaesthesia and were immediately embedded in Tissue Tek OCT compound (Miles Inc., Elkhart, Indiana, USA). Five μm thick cryostat sections were cut from tissue blocks and placed on super frosted slides. Sections were air dried for 3 hours. Slides were wrapped back to back in aluminum foil and stored frozen at -70°C until the time of staining. The rest of the biopsy was fixed in 10% neutral buffered formalin and processed to paraffin blocks. Haematoxylin and eosin (H&E) stained sections were assessed to evaluate the histopathological changes. The extent of inflammatory cells and dermal blood vessels were semi-quantitatively assessed as mild, moderate and severe, compared to the control group.

### Immunohistochemistry

IL-8 utilized in the study is a mouse monoclonal IgG2b antibody raised against a recombinant protein corresponding to amino acids 40–99 mapping at the carboxy terminus of IL-8 of human origin (Santa Cruz, sc 8427). All incubations were done at room temperature.

Prior to staining, sections were brought to room temperature. Tissue sections were fixed in acetone for 10 minutes, air dried and submerged in phosphate buffered saline (PBS) bath for five minutes, before the start of staining. Excess buffer was tapped off followed by the incubation for 60 minutes with the primary antibody diluted at 1:100. Slides were washed twice with PBS for 5 minutes. The Dakocytomation, LSAB 2 was used as detection kit. Biotinylated secondary antibody was applied to the tissue sections for 15 minutes. After washing with PBS, streptavidin was applied for 15 minutes. Slides were washed with PBS then incubated with diaminobenzidine (DAB) chromogen for 10 minutes. Slides were rinsed with distilled water, counterstained with Harris hematoxylin (Hx), dehydrated and finally mounted. Negative controls were slides stained by omission of the primary antibody.

### Staining interpretation

Cytoplasmic staining was considered positive. The intensity of staining was evaluated as 0) negative or absence of positive cells, 1) faint or mild, 2) moderate and 3) strong staining.

### Statistical analysis

Categorical data were compared using Chi-square test and statistical significance was considered at p value < 0.05.

## Results

Sixty two percent of the patients were adolescents. Most of patients were females (82.7%) and 79.3% had positive family history of IAV. Almost half (51.7%) of the patients had a disease duration less than 1 year. The exposure to comedogenic agents was positive in 41.4% and history of medications known to cause acne was seen in 34.5%. The demographic characteristics of the patients are summarized in table 1.

**Table 1:** Clinical characteristics of the study population.

Parameters		Acne (n = 29)	
		
		Frequency	%
Age group in years (p < 0.01)	15 – 20	18	62.1
	21 – 25	7	24.1
	26 – 30	3	10.3
	> 30	1	3.4
Sex (p < 0.01)	male	5	17.2
	female	24	82.8
Family history (p < 0.01)	yes	23	79.3
	no	6	20.7
Duration of disease in years (NS)	< 1 – 2	15	51.8
	> 2 – 5	10	34.4
	> 5	4	13.8
History of exposure to comedogenic agent (NS)	yes	12	41.4
	no	17	58.6
History of medications known to cause acne (NS)	yes	10	34.5
	no	19	65.5
Previous treatment (NS)	local	8	27.6
	systemic	4	13.8
	combined	7	24.1
	no	10	34.5

**Figure 1:**
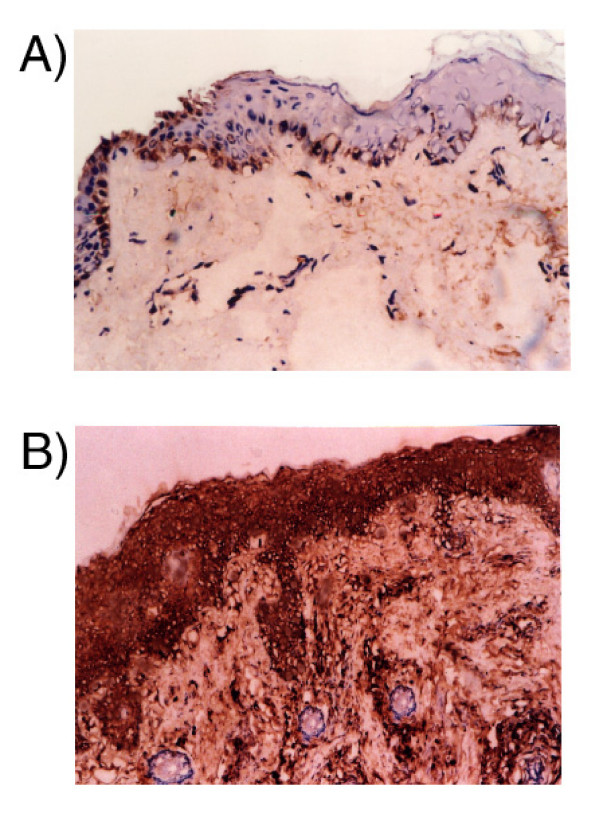
A) Mild staining of epidermal keratinocytes and lack of staining in dermal cells in non lesional skin biopsy. B) Strong cytoplasmic staining of epidermal keratinocytes, dermal endothelial and inflammatory cells. IL-8 IHC, DAB, Hx counterstaining × 40

IHC expression of IL8 was homogenous and confined to the cytoplasm of the cells. It was identified in all IAV patients with variable expressions in the epidermis and dermis ranging from mild to strong. In the epidermis, all keratinocytes were stained. In the dermis the expression was identified in the pilosebaceous units, inflammatory cells and endothelial cells. In the control group only two cases showed mild epidermal expression, while no staining was observed in the remaining cases (figure 1).

Strong association has been found between the extent of epidermal hyperplasia and hyperkeratosis and the epidermal IHC expression of IL-8 (p < 0.001). In addition, the dermal expression significantly paralleled the extent of inflammatory infiltrate (p < 0.001). Moreover, strong association has been found between the extent of dermal expression and the dermal blood vessels (p < 0.001). Furthermore, significant association has been identified between increased dermal IL-8 IHC expression and the progressive disease course (p = 0.02) but not with the duration of the disease. Table 2 illustrates these associations.

**Table 2:** IHC expression of IL- 8 in IAV and control groups

IL-8 expression		Acne		Control	
		
		Frequency	%	Frequency	%
Epidermal P < 0.001	Absent	0	0.0	27	93.1
	Mild	2	6.9	2	6.9
	Moderate	12	41.4	0	0.0
	Strong	15	51.7	0	0.0
Dermal P < 0.001	Absent	0	0.0	0	0.0
	Mild	6	20.6	0	0.0
	Moderate	10	34.5	0	0.0
	Strong	13	44.8	0	0.0

## Discussion

Excessive sebum production secondary to sebaceous gland hyperplasia is the first abnormality to occur in IAV [13]. Subsequent hyperkeratinization of the hair follicle prevents normal shedding of the follicular keratinocytes, obstructing the follicle forming an inapparent microcomedo. Lipids and cellular debris accumulate within the blocked follicle [14]. This microenvironment encourages colonization of P. acne, which provokes an immune response through the production of numerous inflammatory mediators. Inflammation is further enhanced by follicular rupture and subsequent leakage of lipids, bacteria and fatty acids into the dermis.

Data from this study points out to altered immunoreactivity of IL-8 in the lesional skin of acne patients. This was highlighted by the increased expression in IAV skin biopsies compared to normal ones. Increased immunoreactivity was significantly associated with epidermal hyperplasia, follicular hyperkeratosis. In addition, the more pronounced IL-8 expression of the endothelial cells and neutrophilic infiltrate correlated with the extent of dermal inflammatory response and dermal angiogenesis. This expression is concordant with previous studies showing that IL-8 is released by a variety of cell types including monocytes, macrophages, T lymphocytes, fibroblasts, endothelial cells and keratinocytes in response to inflammatory stimulus [4,15,16]. Epidermal hyperplasia and follicular hyperkeratosis secondary to IL-8 production have been noted in psoriatic skin [17]. Early studies on IL-8 point out to its inflammatory nature as a chemokine that attracts and degranulates neutrophils [18,19]. This degranulation release potential key regulators of cell signalling during inflammation such as serine proteases, cathepsin G, leucocyte elastase and proteinase at sites of inflammation, triggering IL-8 and subsequently activate different IL-8 receptors [20].

Contrary to other studies [14,21], IL-8 was mildly expressed in normal keratinocytes in non lesional biopsies in two cases while it was absent in the rest. This may be related to the antibody clone used in the present study. In fact, the pattern of staining was different depending on the clone used in the study of Sticherling et al [15], where suprabasal keratinocytes were staining with 52E8 and all keratinocytes were reactive with 46E5. However, the mild or lack of expression of IL-8 in normal keratinocytes in the present work points to its role in mediating and participating to the inflammatory response in IAV cases.

It has been suggested that local changes in the peripheral blood vessels at the dermal papilla or in the interfollicular region may play a role in evolution of IAV because blood vessels can deliver proinflammatory cytokines that modulated a range of inflammatory and proliferative processes [16]. IL-8 may be a potential endothelial cell growth and survival factor, interacting through its receptors expressed by endothelial cells. The mechanism of IL-8 regulation of angiogenesis may be in part due to enhanced proliferation and survival of endothelial cells by differential expression of antiapoptosis genes and in part by activation of matrix metalloproteinases 2 and 9 [16]. This point is supported in the present study by an increase in endothelial cells IHC expression of IL-8 and increased dermal blood vessels in the skin biopsies of IAV patients.

## Conclusion

We were able to demonstrate the altered immunoreactivity of IL-8 in IAV compared to normal non lesional skin. An important feature of this study is the strong association between IL-8 IHC expression of endothelial cells and dermal inflammatory response and dermal angiogenesis. Targeted therapy to block IL-8 production may hold promise in limiting the deleterious effects of IL-8-mediated inflammatory response and angiogenesis.

## Abbreviations

IAV: inflammatory acne vulgris, IHC immunohistochemisty, IL-8: interleukin 8, P. acnes: propionibacterium acnes

## Competing interests

The author(s) declare that they have no competing interests.

## Authors' contributions

HA carried out the pathological and IHC evaluation and interpretation, participated in drafting and wrote the final manuscript; NS a post graduate student, conducted the data collection and participated in drafting the manuscript. RM participated in drafting the manuscript and MA participated in the design and coordination and helped to draft the manuscript. All authors read and approved the final manuscript.
